# Treatment of Oroantral Fistula in Pediatric Patient using Buccal Fat Pad

**DOI:** 10.5005/jp-journals-10005-1300

**Published:** 2015-08-11

**Authors:** Aviral Agrawal, Ruchi Singhal, Pradeep Kumar, Virendra Singh, Amrish Bhagol

**Affiliations:** Postgraduate Student, Department of Oral and Maxillofacial Surgery, Postgraduate Institute of Dental Sciences, Rohtak, Haryana, India; Senior Resident, Department of Pedodontics, Postgraduate Institute of Dental Sciences, Rohtak, Haryana, India; Postgraduate Student, Department of Oral and Maxillofacial Surgery, Postgraduate Institute of Dental Sciences, Rohtak, Haryana, India; Professor and Head, Department of Oral and Maxillofacial Surgery, Postgraduate Institute of Dental Sciences, Rohtak, Haryana, India; Assistant Professor, Department of Oral and Maxillofacial Surgery, Postgraduate Institute of Dental Sciences, Rohtak, Haryana, India

**Keywords:** Buccal fat pad, Oroantral communication and fistula, Traumatic extraction.

## Abstract

**Brief background:** Oroantral communication (OAC) is the space created between the maxillary sinus and the oral cavity, which, if not treated, will progress to oroantral fistula (OAF). Several methods of surgical OAC repair have been described, but only a few have gained recognition.

**Materials and methods:** A 13 years old male child patient with complaint of difficulty in drinking water and change in voice diagnosed as OAF managed with closure with buccal fat pad (BFP).

**Discussion:** Oroantral fistula is an abnormal communication resulting most frequently from extraction of the upper posterior teeth. Many techniques have been proposed for the closure. The preferred technique may vary from one surgeon to another.

**Conclusion:** The adequate availability of BFP in children, effortless mobilization excellent blood supply and minimal donor site morbidity make it a perfect flap for OAF closure in pediatric patient.

**How to cite this article:** Agrawal A, Singhal R, Kumar P, Singh V, Bhagol A. Treatment of Oroantral Fistula in Pediatric Patient using Buccal Fat Pad. Int J Clin Pediatr Dent 2015;8(2):138-140.

## INTRODUCTION

Oroantral communication (OAC) is the space created between the maxillary sinus and the oral cavity, which, if not treated, will progress to oroantral fistula (OAF) or chronic sinus disease.^1^ The most common precipitating cause of an OAC is the extraction of posterior maxillary teeth, usually the first or second molar. This post-extraction complication occurs more likely if there is preexisting periapical anomaly associated with the offending tooth near the maxillary sinus or extraction of maxillary molar teeth with widely divergent roots. Other causes of OAC include destruction of a portion of the sinus by cysts or benign or malignant tumors, dentoalveolar or implant surgery, etc.^2-4^

Maxillary sinus perforations less than 5 mm close spontaneously after the development of a blood clot in the socket.^5^ Larger openings do not heal spontaneously and necessitate a surgical procedure to close the resulting oroantral opening.

Several methods of surgical OAC repair have been described, but only a few have gained world-wide recognition. In recent years, the use of a pedicled buccal fat pad (BFP) in closure of large oroantral defects has become popular. It was first described by Egyedi^6^ in 1977 for the closure of OAC and oronasal communication.

Because of the smaller volume of the sinus, the risk of the occurrence of OAC in children and adolescents is less. Hereby, we are reporting a case of closure of OAF by BFP in a 13 years old male child which is a rare occurrence.

## CASE REPORT

**Fig. 1:**
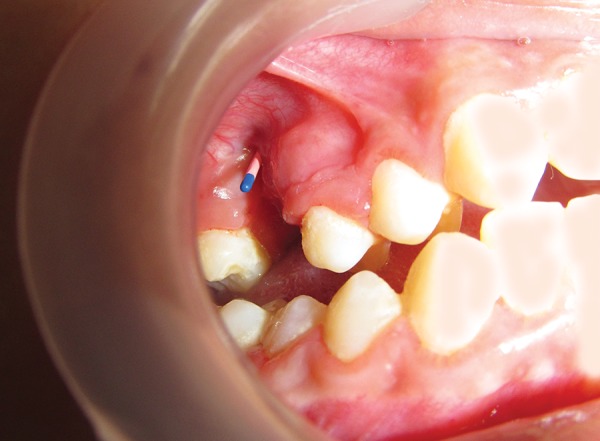
Oroantral fistula with gutta-percha placed in it

**Fig. 2:**
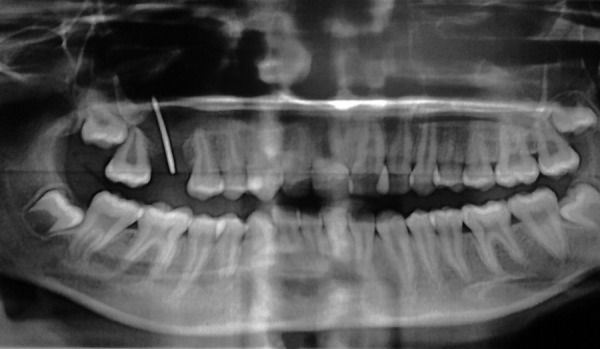
Orthopantomogram showing oroantral defect with gutta-percha placed in it

A 13 years old male child reported to the department of pedodontics, PGIDS, Rohtak with chief complaint of difficulty in drinking water and change in voice since 1 month. Patient gave history of traumatic removal of his right upper back tooth (due to caries) 1 month back following which these problems started. On examination, his right maxillary permanent first molar was missing, there was slight opening on the buccal side of the alveolar process. There was nasal discharge of oral fluids along with nasal twang. Diagnosis of OAF was made (Figs 1 and 2). Patient was then referred to department of oral and maxillofacial surgery for further management.

**Fig. 3:**
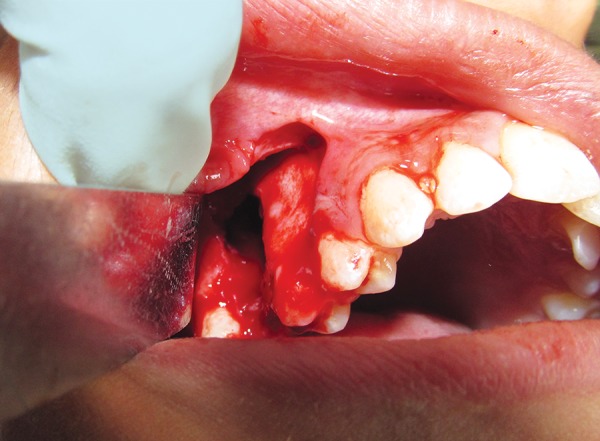
Exposure of the defect

**Fig. 4:**
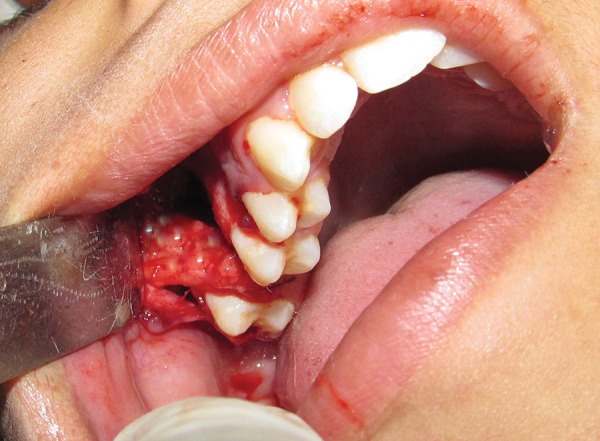
Harvested buccal fat pad positioned over the defect

**Fig. 5:**
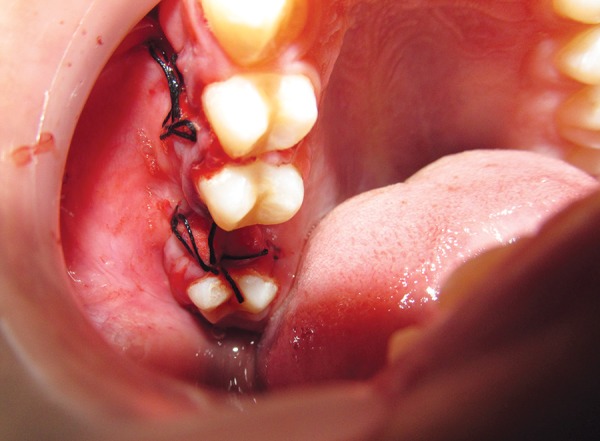
Closure of the defect with buccal fat pad and overlying buccal mucosal flap

**Fig. 6:**
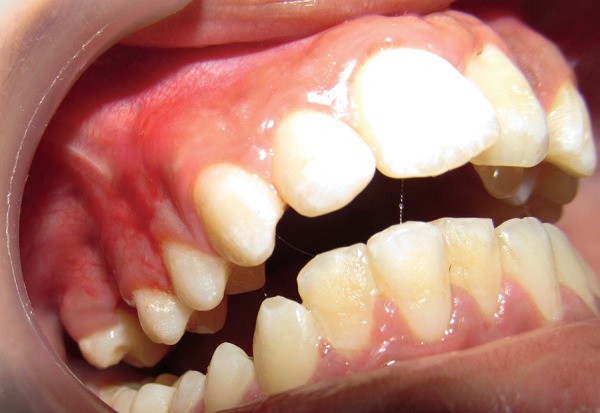
Follow-up photograph showing healed wound

*Management:* His maxillary antrum was irrigated with normal saline three times within a week. After taking informed written consent for the surgery and getting necessary blood investigations, closure with BFP under local anesthesia was planned. Right posterior superior alveolar nerve block and greater palatine nerve block was given with 2% lignocaine with 1:200000 adrenaline along with the right buccal vestibule infiltration. The mucoperiosteal flap was raised from mesial to 15 to distal to 17 with molt’s periosteal elevator after giving incision with no. 15 blade. The defect (Fig. 3) was curetted with the help of Lucas curette. A incision was given over the periosteum on the undersurface of the flap and with pressure applied to the zygomatic arch region, the BFP easily extruded into the operative side. Dissection with a Metzenbaum scissors helped to mobilize as much BFP (Fig. 4) as needed to obtain a tension-free closure across the communication. The palatal mucosa margin was freshened. The fat pad was then sutured with resorbable suture to palatal mucosa. The buccal flap was then positioned over the BFP and was sutured to palatal mucosa with 3-0 silk suture (Fig. 5). Antibiotics and analgesics were prescribed for 5 days. Patient was advised soft diet and to maintain oral hygiene. Sutures were removed after 2 weeks. The postoperative period was uneventful (Fig. 6).

## DISCUSSION

Oroantral communication and subsequent formation of an OAF is a common complication of dental extraction. From a small cavity at birth, the maxillary sinus starts to enlarge during the third month of fetal life and reaches maximum development around the 18 year. The roots of maxillary premolar and molar teeth are in close proximity to the sinus and those of the second premolars and the first molars may be observed within it. The study have shown that removal of the first molars is the most common etiological factor in OAF^7,8^ which was seen in our case.

Review of the literature demonstrates that OAF usually occurs after the third decade of life. Elderly patients with few maxillary teeth appear to have larger sinuses than younger individuals. Because of the smaller volume of OAC the sinus, the risk of the occurrence of in children and adolescents is less. According to few authors,^9^ females exhibit larger sinuses than males and, therefore, are at greater risk of OAF. In the present reported case, the communication was seen in a male child, thus making it an uncommon presentation.

Many techniques have been proposed for the closure of the OF including buccal or palatal flaps and their modifications. The use of some alloplastic materials has also been proposed that ranged from autogenous bone graft^10^ to gold foil.^11^ The preferred technique may vary from one surgeon to another.

Recently, the use of a pedicled BFP in closure of large oroantral defects has become popular. In our case, the OFA was closed with BFP. Anatomically, the BFD has a central body and four processes: buccal, pterygoid, pterygopalatine, and superficial and deep temporal.^12^ The blood supply to the BFP originates from the buccal and deep temporal branches of the maxillary artery, the transverse branch of the superficial temporal artery, and branches of the facial artery. Due to excellent blood supply, the graft has a low failure rate. A possible partial necrosis of the flap has been reported. If the defect is small, spontaneous closure will probably occur. If the area of necrosis is large and fails to close, the other flap techniques should be attempted (such as palatal and tongue). An unusual visible change in facial contour has been reported in patients only when the BFP is used for reconstruction of large defects. In our case no such complication occurred.

Egyedi recommended securing the fat pad with catgut sutures and then covering the graft with a split-thickness skin graft. Tideman et al^13^ used the fat pad as an uncovered pedicled graft with no skin graft and saw complete epithelialization in approximately 2 weeks. They also advised that to diminish the incidence of postoperative complications, the fat pad graft should sufficiently cover the surgical defect and that it should not be sutured under tension. Patient should receive a soft non-chewy diet until healing has taken place. In our case, the fat pad was secured with the vicryl suture and then covered with the buccal flap.

## CONCLUSION

While choosing the surgical treatment of an OAF, its location and size, relation to neighboring teeth, duration, existence of inflamed sinus and the general health status of the patient should be taken into consideration. The adequate availability of BFP in children, effortless mobilization of the BFP and its excellent blood supply and minimal donor site morbidity make it a perfect flap for OAF closure in pediatric patient.

## References

[1] Abuabara A, Cortez AL, Passeri LA, Moraes M, Moreira RWF (2006). Evaluation of different treatments for oroantral/oronasal communications: experience of 112 cases.. Int J Oral Maxillofac Surg.

[2] Guven O (1998). A clinical study on oroantral fistulae.. J Craniomax-illofac Surg.

[3] Babajews A (1986). Multiple myeloma presenting as an oroantral fistula.. Br J Oral Maxillofac Surg.

[4] Hernando J, Gallego L, Junquera L, Villarreal P (2010). Oroantral communications: a retrospective analysis.. Med Oral Patol Oral Cir Bucal.

[5] von Wowern N (1973). Correlation between the development of an oroantral fistula and the size of the corresponding bony defect.. J Oral Surg.

[6] Egyedi P (1977). Utilization of the buccal fat pad for closure of oroantral and/or oro-nasal communication.. J Maxillofac Surg.

[7] Killey HC, Kay LW (1967). An analysis of 250 cases of oroantral fistula treated by the buccal flap operation.. Oral Surg.

[8] Ehrl PA (1980). Oroantral communication.. Int J Oral Surg.

[9] Lin PT, Bukachevsky R, Blake M (1991). Management of odontogenic sinusitis with persistent oroantral fistula.. Ear Nose and Throat.

[10] Proctor B (1969). Bone graft closure of larger or persistent oromaxil-lary fistula.. Laryngoscope.

[11] Goldman EH, Stratigos GT, Arthur AL (1969). Treatment of oroantral fistula by gold foil closure.. J Oral Surgery.

[12] Arce K (2007). Buccal fat pad in maxillary reconstruction.. Atlas Oral Maxillofac Surg Clin North Am.

[13] Tideman H, Bosanquet A, Scott J (1986). Use of the buccal fat pad as a pedicled graft.. J Oral Maxillofac Surg.

